# Multi-institutional evaluation comparing guidance from International Ki67 Working Group vs National Health Commission of China on immunohistochemistry-based Ki67 assessment alongside the Quantitative Dot Blot method

**DOI:** 10.3389/fonc.2024.1510273

**Published:** 2025-01-27

**Authors:** Yin Wang, Jiarui Zou, Qinghua Cao, Guihong Dai, Panhong Fan, Xue Gong, Jinyan Jiang, Yanqing Kong, Chao Liu, Chunhui Liu, Chenjia Lu, Meiren Li, Zhiqiang Lang, Yang Lin, Yan Peng, Haiyan Shi, Yuhuan Wang, Jiu Wang, Bichen Xie, Bing Yang, Guohua Yu, Cuiping Zhang, Hengming Zhang, Luting Zhou, Zilan Zhang, Zhenli Zhu, Junmei Hao

**Affiliations:** ^1^ Department of Pathology, Yantai Affiliated Hospital of Binzhou Medical University, Yantai, China; ^2^ Department of Pathology, Hangzhou Red Cross Hospital, Hangzhou, China; ^3^ Department of Pathology, The Affiliated Taizhou People’s Hospital of Nanjing Medical University, Taizhou School of Clinical Medicine, Nanjing Medical University, Taizhou, China; ^4^ Department of Pathology, Henan Provincial People’s Hospital, Zhengzhou, China; ^5^ Department of Pathology, Qinghai University Affiliated Hospital, Xining, China; ^6^ Department of Pathology, Chenzhou No. 1 People Hospital, Chenzhou, China; ^7^ Department of Pathology, Shenzhen Maternity and Child Healthcare Hospital, Shenzhen, China; ^8^ Department of Pathology, Guangdong Provincial People’s Hospital (Guangdong Academy of Medical Sciences), Southern Medical University, Guangzhou, China; ^9^ Department of Pathology, The People’s Hospital of LongHua, Shenzhen, China; ^10^ Department of Pathology, Jiujiang University Affiliated Hospital, Jiujiang, China; ^11^ Department of Pathology, Affiliated Yantai Yuhuangding Hospital, Qingdao University, Yantai, China; ^12^ Department of Health statistics, School of Public Health, Binzhou Medical University, Yantai, China; ^13^ Department of Pathology, The Third Affiliated Hospital of Soochow University/Changzhou First People’s Hospital, Changzhou, China; ^14^ Department of Pathology, Guangdong Hospital of Integrated Traditional Chinese and Western Medicine, Guangzhou, China; ^15^ Department of Pathology, People’s Hospital of Xinjiang Uygur Autonomous Region, Urymqi, China; ^16^ Department of Pathology, Affiliated Hospital of Jiangnan University, Wuxi, China; ^17^ Department of Pathology, Weifang People’s Hospital, Weifang, China; ^18^ Department of Pathology, Ruijin Hospital, Shanghai Jiaotong University School of Medicine, Shanghai, China; ^19^ Department of Pathology, Northern Jiangsu People’s Hospital, Yangzhou, China

**Keywords:** breast cancer, Ki67, QDB, IHC, IKWG, NHCC

## Abstract

**Purpose:**

Recommendations from the National Health Commission of China (NHCC) and the International Ki67 Working Group (IKWG) were issued to guide immunohistochemistry (IHC)-based Ki67 scoring for breast cancer patients in daily clinical practice. They were evaluated in this multi-institutional study alongside the results from the Quantitative Dot Blot (QDB) method.

**Methods:**

Three alternative adjacent sections from 40 primary ER+ breast cancer resection blocks were randomly assigned a number from 1 to 120 for Ki67 staining and reviewed by 21 pathologists, while the other three alternative sections were sent for QDB analysis of Ki67 protein levels. Ki67 scores were grouped by 5/30% (IKWG), 10/30% (NHCC) and 20/30% (NHCC appendix 9, NHCCa9), respectively while QDB results were grouped by C_5_–C_95_ of 2.31 nmol/g defined in previous study as low-, equivocal-, and high-risk groups.

**Results:**

The overall Intraclass Correlation Coefficient (ICC) was 0.785 for IHC evaluations from 21 pathologists, with Fleiss Kappa values of 0.555, 0.628, and 0.480 when Ki67 scores were grouped by guidance from IKWG, NHCC, and NHCCa9, respectively. In comparison, the ICC and Fleiss kappa values for the QDB analysis were 0.939 and 0.831, respectively. When IHC and QDB results were cross-referenced, more specimens were grouped as high-risk by QDB than IHC, and NHCCa9 led to the highest percentage of disagreement between the two methods.

**Conclusion:**

The IKWG recommendation was harder to achieve categorized agreement among pathologists than the NHCC recommendation, yet it led to the best agreement with the QDB to define the low-risk group. The QDB method offers significantly improved consistency compared to the current IHC-based Ki67 assessment.

## Introduction

The nuclear proliferation biomarker Ki67 may be one of the most commonly used protein diagnostic biomarkers for all types of cancer (1). Its expression level is widely regarded as a reflection of tumor aggressiveness. For breast cancer patients, it is also critical to consider the benefits of chemotherapy in daily clinical practice worldwide. Thus, it is required for every new breast cancer patient by the National Health Committee of China (NHCC), according to its latest guidance (2, 3).

The current Ki67 assessment method relies on immunohistochemistry (IHC) in daily clinical practice. The percentage of positively stained nuclei, or Ki67 score, was evaluated in the tumor tissue to reflect the aggressiveness of the tumor. However, this method is clearly far from satisfactory in real-world practice, as intensive efforts have been launched aiming to amend issues associated with this method over the years (1, 2, 4).

The International Ki67 Working Group, or IKWG, suggested that “*In this T1-2, N0-1 patient group, the IKWG consensus is that Ki67 5% or less, or 30% or more, can be used to estimate prognosis*” for identifying estrogen receptor (ER) positive and Her2 negative breast cancer patients who may not need adjuvant chemotherapy (2). The NHCC guidance issued in 2022, on the other hand, considered Ki67 scored between 10% and 30% as borderline samples, requiring evaluation of more than 500 invasive breast cancer cells to improve the consistency of the results (3). However, under the same guidance, Appendix 9 (NHCCa9), the guidance suggested that between pathological laboratories, the scores between 20% and 30% should be used as cutoffs to determine the necessity of adjuvant chemotherapy for breast cancer patients.

In all these guidelines, the Ki67 scores were categorized as low, equivocal, and high-risk groups, with the equivocal group varying between 5%–30% (IKWG), 10%–30% (NHCC), and 20%–30% (NHCCa9). In this study, three recommendations were compared by inviting 21 pathologists from 18 hospitals across China to evaluate the same set of ER-positive breast cancer resections.

Meanwhile, we have demonstrated in a series of studies that absolute quantitation of Ki67 from formalin fixed paraffin-embedded (FFPE) specimens using the Quantitative Dot Blot (QDB) method may be used to identify ER-positive patients for adjuvant chemotherapy (5, 6). A putative cutoff of 2.31 nmol/g was developed based on overall survival (OS) analysis and independently validated using another cohort of breast cancer specimens (6).

Unlike IHC, QDB is an objective and quantitative biochemical assay. Accordingly, we categorized the QDB results into low-, equivocal-, and high-risk groups based on the C_5_ and C_95_ values of the defined 2.31 nmol/g cutoff as a reflection of the reliability of the assay. In other words, the low risk was defined as ≤C_5_, the equivocal group were between C_5_ and C_95,_ and the high risk as ≥C_95_ of 2.31 nmol/g (the process of identification of C_5_ and C_95_ of the cutoff was included in the *Materials and methods* section). Clearly, unlike the cases of IKWG, NHCC, and NHCCa9, this categorization is based on statistical analysis to minimize the influence of random error.

In this study, an organizer (Hao) chose 40 ER+ breast cancer tissue surgical resection blocks for IHC and QDB analyses side by side. Three adjacent slices from each block were used for IHC analysis and scanned for online accessibility. The organizer assigned a number from 1 to 120 randomly to each of these 120 IHC slides, without revealing to the invited pathologists that there were triplicates for each block until after the completion of the evaluation. The Ki67 scores were categorized as low, equivocal, and high-risk groups by following IKWG, NHCC, and NHCCa9 guidance, respectively, to evaluate the practicability of these three guidelines, as well as those of the QDB method when categorized using C_5_ and C_95_ of the defined 2.31 nmol/g in previous studies using the protein lysates prepared from three alternative adjacent slices of each block.

## Results

The pathological characteristics of the 40 breast cancer surgical resection blocks are shown in **Table 1**. These patients were all ER-positive, with the majority also PR-positive (37 vs 3). More patients were over 50 years of age (25 years vs 15 years). All lymph node statuses were included, as were all pathological tumor sizes and histological grades. However, more patients were at pT1 and pT2 for tumor size and pN0 and pN1 for lymph node status. The majority were histologically grade II (29 of 40). As expected, the majority of the patients were Her2− (37 vs 3).

**Table 1:** Clinicopathological characteristics of all 40 luminal breast cancer patients included in the current study.

Characteristics	Number of Cases (%)
Age	
<50	15 (37.5)
≥50	25 (62.5)
Pathological Lymph Node Status, pN	
pN0	22(55.0)
pN1	10(25.0)
pN2	5(12.5)
pN3	3(7.5)
pathological Tumor Size, pT	
pT1	17(42.5)
pT2	20(50.0)
pT3	1(2.5)
pT4	2(5.0)
Histological Grade	
**I**	1(2.5)
**II**	29(72.5)
**III**	7(17.5)
Unknown	3(7.5)
ER	
≥1%	40(100.0)
PR	
<1%	3(7.5)
≥1%	37(92.5)
HER2	
HER2-	37(92.5)
HER2+	3(7.5)

**Figure 1:**
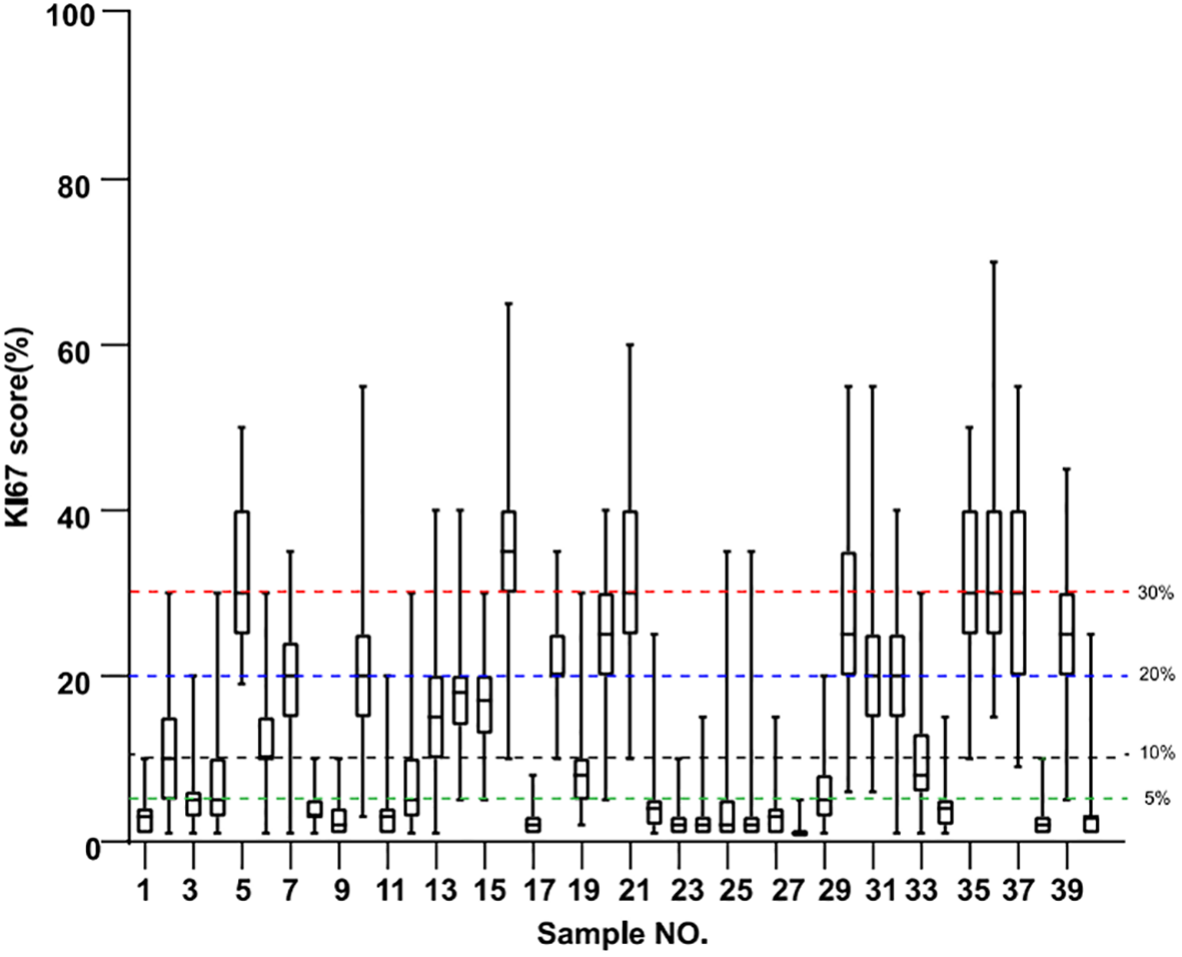
Boxplots of Ki67 evaluation of 40 samples by 21 pathologists. Resection blocks from each specimen were serially sectioned, and the 2nd, 4th, and 6th sections were used for IHC analysis. The green line indicates the suggested 5% cutoff by the IKWG, the black line indicates the suggested 10% cutoff by the NHCC, the blue line indicates the suggested 20% cutoff by NHCC appendixa9, and the red line indicates the 30% cutoff shared by all three guidelines.

The Ki67 scores for all 40 specimens from the 21 pathologists are shown in the boxplot in **Figure 1**. Cutoffs used in the three guidelines were indicated by lines of different colors, with green indicating the 5% cutoff from IKWG, black for the 10% cutoff from NHCC, blue for the 20% cutoff from NHCCa9, and red for the 30% cutoff shared by all three guidelines to identify specimens in the high-risk group. The detailed scores are also reported in **Table 2**, with triplicated Ki67 scores for each block listed within the same cell. As shown in **Figure 1**, only one out of 40 specimens achieved 100% agreement as low risk when IKWG guidance was followed. This number reached seven with NHCC guidance and 13 with NHCCa9 guidance. However, none of the specimens achieved 100% agreement at a high risk in this study.

**Table 2:** Ki67 scores from all 21 pathologists colored under IKWG guidance.

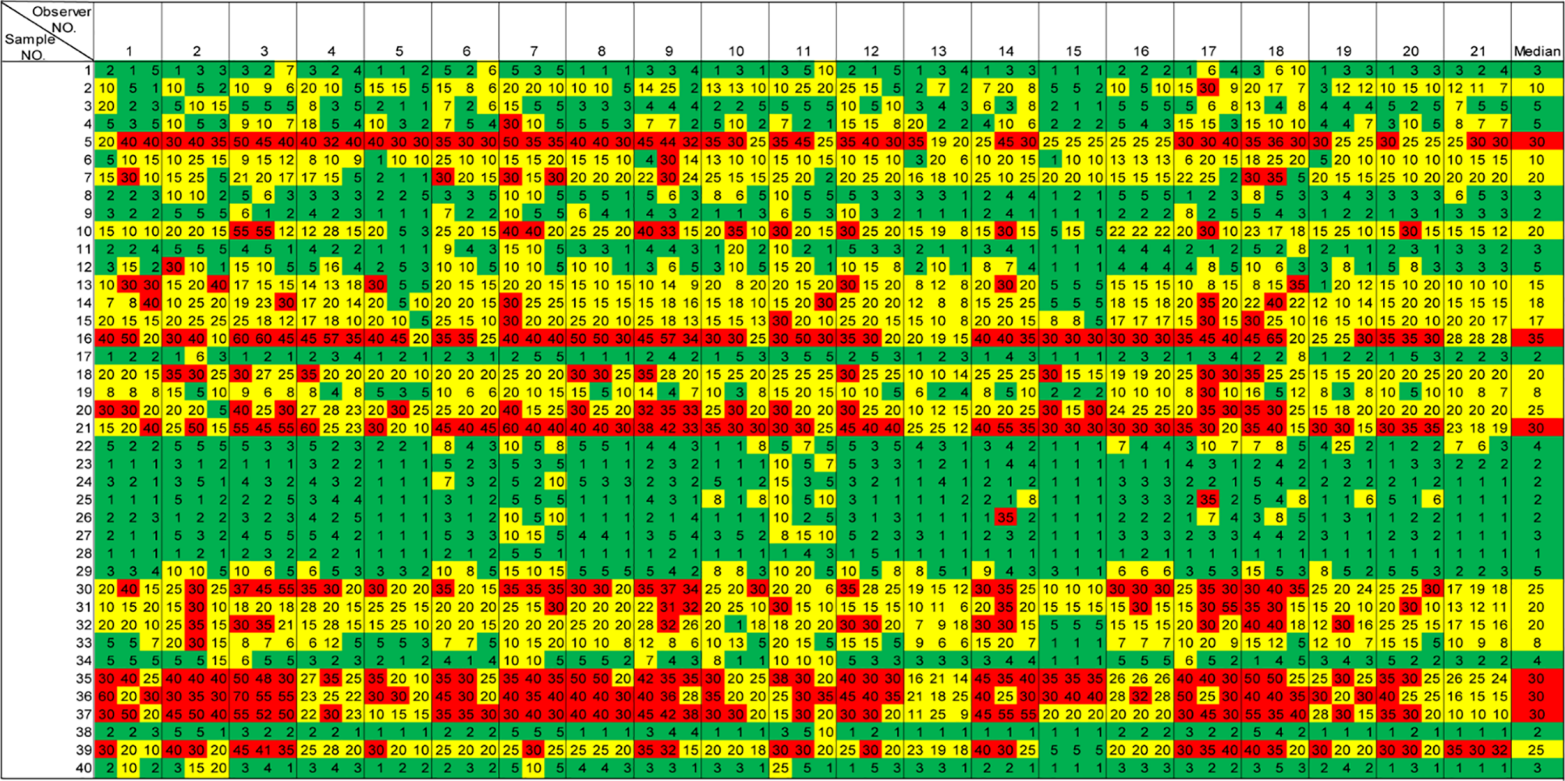

The three numbers within each row of 21 observers represent the observations of three alternative adjacent slices made by the same observer in the study. Numbers are highlighted in green to indicate a value ≤5%, in yellow to indicate a value between 5% and 30%, and in red to indicate a value ≥30%.

In **Table 2**, a heat map was generated based on the IKWG guidance, with ≤5% as green for the low-risk group, between 5% and 30% in yellow for the equivocal group, and ≥30% in red for the high-risk group. In **Supplementary Tables 1A, B**, the same set of colors were applied to indicate low-, equivocal-, and high-risk groups based on NHCC and NHCCa9 guidance.

The categorized consistency of IHC was analyzed using Fleiss Kappa analysis. We found that NHCC had the highest overall Kappa value of 0.628 (95%CI: 0.628–0.629). IKWG guidance led to an overall Kappa value of 0.555 (95%CI: 0.554–0.555), and the NHCCa9 guidance led to the lowest Kappa value of 0.480 (95%CI: 0.479–0.481).

**Figure 2:**
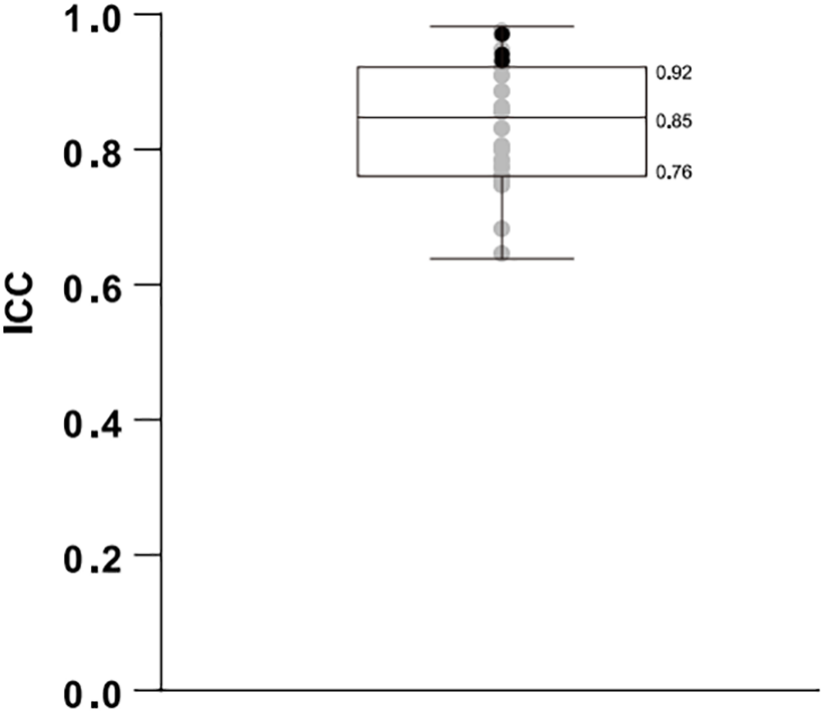
Boxplots of the intra-rater intraclass correlation coefficients (ICC) from 21 pathologists and the three technicians performing QDB analysis are plotted together. The gray dots represent the ICC from pathologists, whereas the black dots represent those from technicians.

The inter-rater intraclass correlation coefficient (ICC) was 0.785 (95%CI, 0.708–0.858). The intra-rater ICC was also investigated among the invited pathologists. It should be emphasized that the reading of the triplicate section from each sample was blinded to all but the organizer of the study. We found that the single measurement of ICC ranged from 0.639 to 0.982, with 25% percentile at 0.76, a median of 0.848 and 75% percentile at 0.9225 (**Figure 2**).

**Figure 3:**
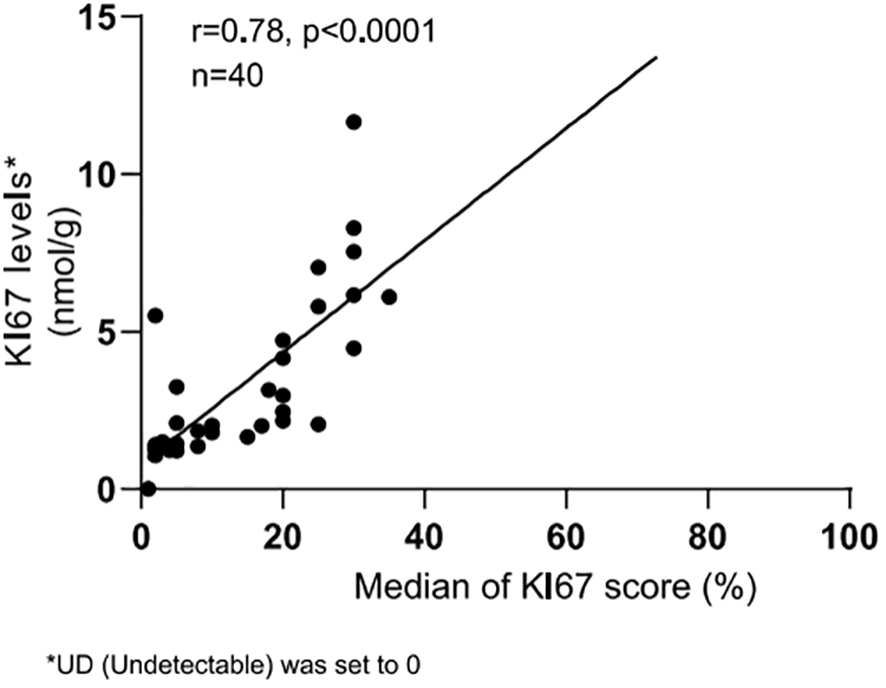
Correlation between Ki67 scores from IHC analysis and Ki67 levels from QDB measurements. The median Ki67 scores from all pathologists for each breast cancer sample were plotted against the averaged Ki67 levels from all QDB measurements of each individual sample using Pearson’s correlation analysis. R = 0.78, indicating a strong correlation between the QDB results and IHC analysis. Ki67 levels were arbitrarily set to 0 nmol/g for samples with background Ki67 levels (UD).

Three alternative adjacent slices were also used for the QDB analysis, as shown in **Table 3**. The lysates were analyzed by three technicians, each measured in triplicate for three times. The overall CV of all the specimens was 15.86%. When plotted against the median Ki67 scores from 21 pathologists, the QDB results were highly correlated with those of the IHC assessment, with r = 0.78, p <0.0001 using Pearson’s correlation analysis (**Figure 3**).

**Table 3:** Ki67 levels of three technicians by measuring the total protein lysates prepared from three alternative adjacent FFPE slices three times independently, each in triplicate.

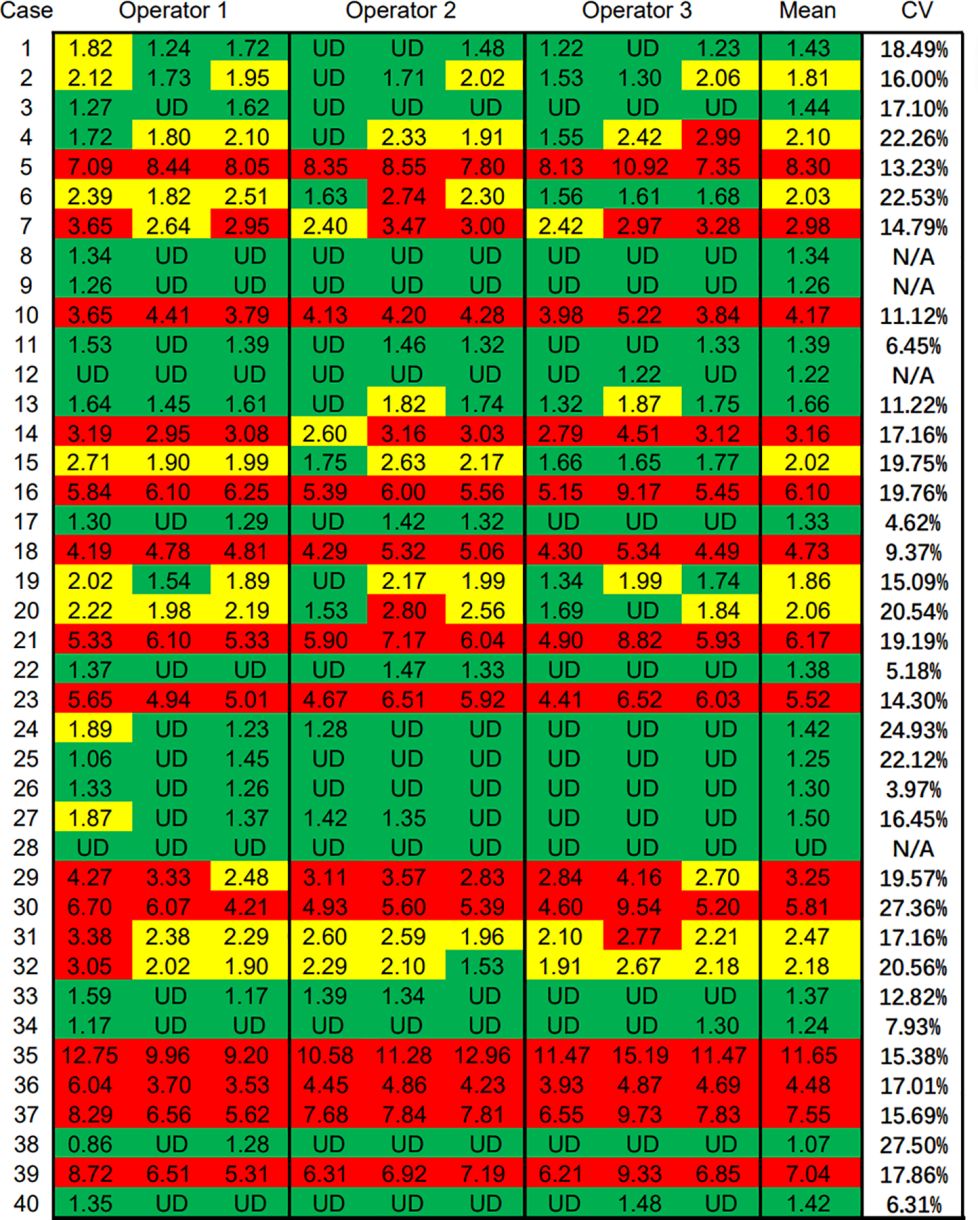

The numbers were categorized into different risk groups based on the C_5_ and C_95_ of the QDB assay, with ≤1.793 nmol/g in the low-risk group in green, between 1.793 nmol/g and 2.727 nmol/g in the equivocal group in yellow, and ≥2.727 nmol/g in the high-risk group in red. Ki67 level less than 25 pg (about 1.4 nmol/g) was defined as Limit of Quantitation and noted undetectable level (UD). The mean and CV of each sample were calculated based on the Ki67 levels from nine assays for each sample, as indicated in the figure, with an overall CV of 15.86%. For samples with the majority as UD, the CV was not calculated and was reported as N/A.

We also performed C_50_ studies to determine the C_5_ and C_95_ of 2.31 nmol/g at 1.793 nmol/g and 2.727 nmol/g, respectively (**Supplementary Figure 1**). The Ki67 levels from QDB analysis were thus categorized as ≤C_5_ as the low-risk group in green, between C_5_ and C_95_ as the equivocal group in yellow, and ≥C_95_ as the high-risk group in red (**Table 3**).

The overall ICC of QDB was 0.939 (95%CI: 0.908–0.963), which was significantly higher than that of IHC. The categorized consistency of the QDB method was also significantly higher than that of the IHC-based method, with an overall Fleiss Kappa of 0.831 (95%CI: 0.827–0.836). The intra-rater ICC for the three technicians was calculated at 0.924, 0.933, and 0.963, respectively. As shown in **Figure 2**, all intra-rater ICCs of the QDB analysis were above the 75% percentile of those of the IHC analysis.

When we compared the categorized QDB results with those of IHC, we observed that there were more specimens categorized as high-risk by the QDB method than by the IHC method (14/40 vs 6/40) (**Table 4**). The discordant specimens categorized as high-risk by the QDB method were more likely to be categorized as the equivocal group by IKWG and NHCC guidelines, but as the low-risk group by the NHCCa9 guideline. Unexpectedly, we identified two specimens (#23 and #29) grouped as high-risk by the QDB method, but as low-risk by any of the three IHC guidelines. Overall, we found that Ki67 scores tended to be conservative when evaluated using the IHC method compared to those evaluated using the QDB method.

**Table 4:** Evaluation of the consistency of Ki67 evaluation by IHC vs QDB. The mean Ki67 levels of each breast cancer specimen measured by QDB assays were categorized into low-, equivocal-, and high-risk groups based on C_5_ and C_95_ of the assay.

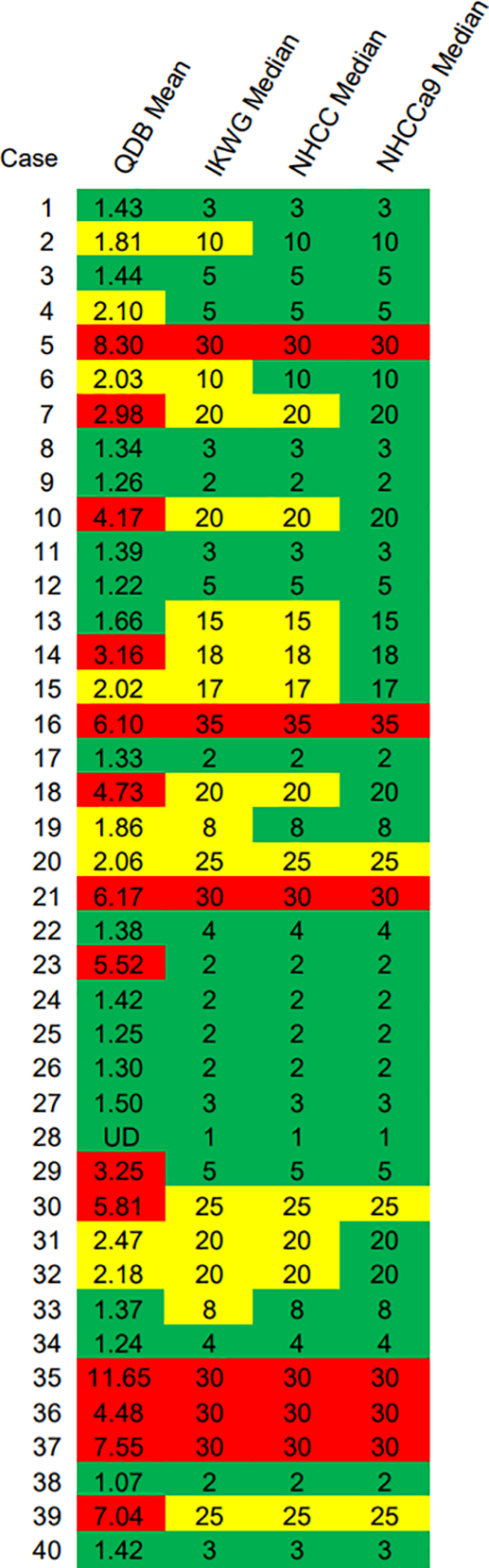

The median Ki67 scores from the 21 pathologists were also categorized into low-, equivocal-, and high-risk groups based on the three guidelines. All those categorized as low-risk are highlighted in green, equivocal in yellow, and high-risk in red.

One of the major goals of Ki67 assessment is to identify patients who may be spared of chemotherapy, i.e., those in the low-risk group. There were 19 specimens in the low-risk group as determined by IKWG, 23 by NHCC, and 31 by NHCCa9. When the QDB results were used as a reference, we found 84.21% (16/19), 73.91% (17/23), and 58.06% (18/31) agreement with IKWG, NHCC, and NHCCa9, respectively (**Table 4**). In other words, QDB results were in the best agreement with IKWG and worst with NHCCa9.

## Discussion

In this study, by inviting 21 pathologists to assess Ki67 scores of the same set of luminal-like breast cancer specimens, we were able to evaluate the practicability of three guidelines (IKWG, NHCC, and NHCCa9), as well as that of Quantitative Dot Blot (QDB)-based Ki67, in daily clinical practice.

Our results demonstrated that consistency is easier to achieve among pathologists by following NHCC guideline, whereas it is harder to achieve by NHCCa9 guideline. However, if QDB results are used as a reference, the IKWG guideline offers the best guidance to identify patients in the low-risk group, i.e., those spared of chemotherapy, while NHCCa9 offers significantly more false-negative results.

We were also able to investigate the intra-rater ICC by assigning random numbers from 1 to 120 to the alternative adjacent triplicate sections of the 40 samples. We believe that this design will best reveal the potential subjectivity of IHC analysis in real-world practice. Admittedly, a few pathologists may be aware of repeated images during the evaluation. However, even if this situation existed, it had a minimal impact on the overall assessment.

The intra-rater ICC was calculated to be as low as 0.639, with 25% percentile at 0.76, median at 0.848 and 75% percentile at 0.9225. We interpreted that even for an experienced pathologist in China, there was only an 85% chance on average to score the same IHC slide consistently.

This study was limited to the evaluation of a set of pre-stained slides. Thus, we were unable to evaluate the potential variations associated with preanalytical factors in individual institutions. All invited pathologists were not through extensive training, in addition to broad instruction. Thus, we believe that this study faithfully reflects real-world practice for all invited pathologists.

We were surprised to find that there were 20% (8/40) specimens categorized as the equivocal group based on the C_5_–C_95_ of the purposed 2.31 nmol/g identified in a previous study using the QDB method. We interpreted that the precision of the QDB method remains to be improved, as its improvement should further narrow the window of the equivocal group in the future. It should also be noted that the proposed 2.31 nmol/g remained to be validated in the future with a much larger scale of study. However, we expect that the possible adjustment of this cut-off should have a minimum impact on the overall conclusion.

One unexpected observation was that while the overall agreement between the QDB and IHC methods was satisfactory (r = 0.78 by Pearson), there were clear differences between the two specimens, #23 and #29. They were grouped as a high-risk group by the QDB method, but as a low-risk group by the IHC method according to all three guidelines. One possible explanation may be the negative influence of heavy counterstaining on the nuclear antigen, especially for #23, as suggested by Rudbeck (7). Another possibility is incorrect staining due to poor pre-staining treatment. However, this point was debatable, even among the invited pathologists. The IHC images of these two specimens, as well as other representative IHC images from other specimens, are provided in the supplemental data (**Supplementary Figure 2**), warranting further discussion of this clear discrepancy between the two methods.

It should also be pointed out that there was a difference in the nature of the results from the QDB analysis and IHC analysis. In the QDB, the total protein lysates were extracted from FFPE slices by disrupting of the tissue structure. Thus, QDB measures the average protein content to minimize the heterogeneity of the tissue slice. In contrast, Ki67 scores reflect the localized Ki67 protein level with a fully preserved tissue structure, thus better reflecting the heterogeneity of the tissue slice. The results from these two methods should be highly correlated, but not identical, as demonstrated by the current study.

It is unclear which method would provide more relevant results for the prognosis and prediction of patients. While some argue that tissue heterogeneity might be better reflected through IHC analysis, it is arguable that the QDB method might maximally minimize the negative influence of tissue heterogeneity on the prognosis and prediction of patients. Clearly, final judgement may only be achieved through properly controlled prospective clinical trials in the future.

One limitation of the current study is that we invited 21 pathologists for IHC analysis; however, only three technicians were requested for QDB analysis. The limited number of technicians for the QDB analysis may underestimate the variations among technicians when interpreting the QDB results. In contrast, QDB analysis is an objective biochemical assay that is tightly controlled. The C_5_/C_95_ analysis also fully considers the variations among technicians in a large scale. Thus, we interpreted that the potential impact of including more technicians in the QDB analysis should not fundamentally change the overall conclusion of the current study.

Another limitation of the current study is that IKWG recommends a global scoring method, whereas NHCC prefers the hotspot method. In the current study, we purposely selected resection blocks with no hotspots only appearing at the junction of tumors and normal tissues, while the Ki67 was low within the tumor to avoid potential issues of methodological differences; thus, we were able to achieve better agreement between these two methods.

In conclusion, by inviting 21 experienced pathologists to score the Ki67 levels of the same set of IHC slides from 40 ER+ breast cancer specimens, we were able to compare the practicability of the three clinical guidelines (IKWG, NHCC, and NHCCa9) in daily clinical practice. We were also able to compare the Ki67 scores with results from QDB measurements to suggest that QDB may significantly improve the consistency of Ki67 assessment in daily clinical practice. Our results also showed that if QDB results are used as a reference, the IKWG guide has difficulty achieving agreement among pathologists, yet provides the most trustworthy guide for chemotherapy for luminal-like patients.

## Materials and method

### Human subjects

The inclusion criteria were patients diagnosed with invasive breast cancer with FFPE resection specimens available at the Yantai Affiliated Hospital of Binzhou Medical University, Yantai, PR China, between 2015 and 2017. The specimens must be resection blocks with ER+ based on IHC analysis and have more than 50% tumor tissues based on H&E staining. All studies were performed in accordance with the Declaration of Helsinki and were approved by the Medical Ethics Committee of Yantai Affiliated Hospital of Binzhou Medical University (Approval # 20191127001 to Dr. Hao), and informed consent forms were waived for archived specimens.

### Sample preparation and distribution

For each of 40 resection blocks, seven adjacent sections were prepared, with the 1st stained with H&E, the 2nd, 4th, and 6th stained with IHC method using MIB1 antibody against Ki67. The 3rd, 5th, and 7th sections were used to extract total tissue lysates for QDB measurement using the same MIB1 antibody against Ki67.

The 120 IHC-stained slides were randomly assigned number 1 to 120, and sent out for scoring using the NHCC guideline stated as the following “*Our recommendation is that the whole slice be evaluated under a low-power field to determine whether the positive cells are uniformly distributed. If positive cells were uniformly distributed, three or more high-power fields were randomly counted, and an average Ki-67 index was obtained. If the positive cells are unevenly distributed, a prominent “hot spot” of Ki-67 index may exist. If a hotspot appears at the junction of tumors and normal tissues and the Ki-67 index is relatively low within the tumor, it is recommended that the Ki-67 index in three or more high-power fields should be counted in the tumor margin area. If a hot spot appears within the tumor, the Ki-67 index of the whole slice can be evaluated on an average of three or more high-power fields including the hotspot area should be selected. When the Ki-67 index is within the critical range of 10%−30%, it is recommended that more than 500 invasive carcinoma cells should be evaluated as much as possible to improve the accuracy* (3)*.”* Because all specimens were resection blocks, the study coordinator (Hao) screened the entire set of slides to ensure that no hotspots only appeared at the junction of the tumor and normal tissues, whereas Ki67 was low within the tumor. Thus, all invited pathologists were encouraged to follow the IKWG recommendations to minimize discrepancies in interpreting the image. There have been no attempts initiated to standardize the scoring method other than the guidance. Variations in scoring among the participants were expected.

All the invited pathologists were certified pathologists with a minimum of 10 years of experience in the hospital. The scoring process was blinded to all participants, except for the study coordinator (Hao). All invited participants considered the 120 IHC slides as independent sections and had no prior knowledge of the QDB results until after submitting the Ki67 scores.

All 120 IHC slides were scanned digitally. The IHC slides are available for evaluation during and after the publication of the manuscript upon written request from Dr. Junmei Hao.

### General reagents

Mouse anti-Ki67 antibody (clone MIB1) was purchased from ZSGB-BIO (Beijing, China). HRP-labeled Donkey Anti-Mouse IgG secondary antibody was purchased from Jackson ImmunoResearch Lab (Pike West Grove, PA, USA). All other chemicals were purchased from Sinopharm Chemicals (Beijing, PR China). The KI67 recombinant protein was prepared by Quanticision Diagnostics, Inc., and the preparation method has been previously published (5).

### QDB analysis

All QDB analyses were performed by Quanticision Diagnostics Inc. The detailed method has been described elsewhere (5, 6). Briefly, sections of all 40 breast cancer specimens were used to extract total protein lysates. Total of 0.5 μg was loaded into a QDB plate together with serially diluted recombinant KI67 purified protein in triplicate. The loaded QDB plate was dried for 4 h at RT and then blocked in 4% non-fat milk for 1 h. Anti-Ki67 antibody (MIB1) was diluted 1:1,000 in blocking buffer, incubated with QDB plate at 100 μl/well overnight at 4°C, and incubated with a donkey anti-mouse secondary antibody (diluted 1:2,500 in blocking buffer) on a shaker at 100 rpm for 4 h at RT. After the last wash, the QDB plate was inverted for 1 min and the TBST waste liquid was extracted using a filter pump. The QDB plate was inserted into a white 96-well plate pre-filled with 100 μl/well ECL working solution for 3 min for quantification with Tecan Infiniti 200pro Microplate reader with the option “plate with cover.”

The consistency of the experiments was ensured by including 293T cell lysates with known Ki67 levels in all experiments. The result was considered valid when the calculated Ki67 level of 293T was within 20% of known Ki67 level at 12.5 (10.0–15.1) nmol/g, respectively. Absolute Ki67 levels were determined based on the dose curve of the protein standard. Ki67 level of less than 25 pg (approximately 1.4 nmol/g) was defined as the Limit of Quantitation and an undetectable level (UD).

QDB analysis was performed by three technicians using the same set of total protein lysates in triplicate, and the experiments were repeated three times for nine independent measurements of Ki67 levels in the 40 breast cancer specimens.

### Defining C_5_ and C_95_ of Ki67 cutoff

Multiple breast cancer specimens were screened to identify those with Ki67 levels ≥5 nmol/g. Lysates from three specimens were mixed and serially diluted at 1:1.3 until at 0.81 nmol/g, supplemented with 0.25 μg/μl IgG free BSA. The prepared lysates were loaded to QDB plate as 56-plicates at each dose, and the Ki67 levels were measured through QDB analysis. The number of samples at each dose with their Ki67 values above 2.31 nmol/g were used to calculate C_5_, C_50_, and C_95_ using the probit model of SPSS software at 1.793 nmol/g, 2.26 nmol/g, and 2.727 nmol/g, respectively. This experiment was performed twice, with highly consistent results.

### Statistical analysis

All data were analyzed using SPSS 26.0. The overall agreement of the Ki67 scores from 21 pathologists with three independent evaluations of each specimen, as well as that of QDB results from three technicians with three independent measurements of each specimen, was assessed using the Intraclass Correlation Coefficient (ICC) method. The inter- and intra-personal agreement of the Ki67 scores from three independent evaluations of each of the 40 specimens by 21 pathologists, as well as those of the Ki67 levels from three independent measurements of all three technicians, were also analyzed using ICC.

The Ki67 scores from 21 pathologists were also categorized into low-, equivocal-, and high-risk groups based on the guidelines of the International Ki67 Working Group (IKWG), National Health Committee of the People’s Republic of China (NHCC), and National Health Committee of the People’s Republic of China (Appendix 9, NHCCa9). The overall performance of each guideline was assessed using the Fleiss Kappa test. The Ki67 levels from QDB measurements were also categorized as low-, equivocal-, and high-risk groups based on the C_5_ and C_95_ of the 2.31 nmol/g cutoff defined in previous studies, and the consistency of QDB measurements from three technicians was also assessed using the Fleiss Kappa test.

## Data Availability

The original contributions presented in the study are included in the article/**Supplementary Material**. Further inquiries can be directed to the corresponding author.
