# Supplementary material for: Multi-institutional evaluation comparing guidance from International Ki67 Working Group vs National Health Commission of China on immunohistochemistry-based Ki67 assessment alongside the Quantitative Dot Blot method
Source: Front Oncol. 2025 Jan 27;14:1510273. doi: 10.3389/fonc.2024.1510273 (PMC11808281; doi:10.3389/fonc.2024.1510273)
Supplement: Supplementary file 1 [file DataSheet1.docx]

**Supplementary figures and tables**


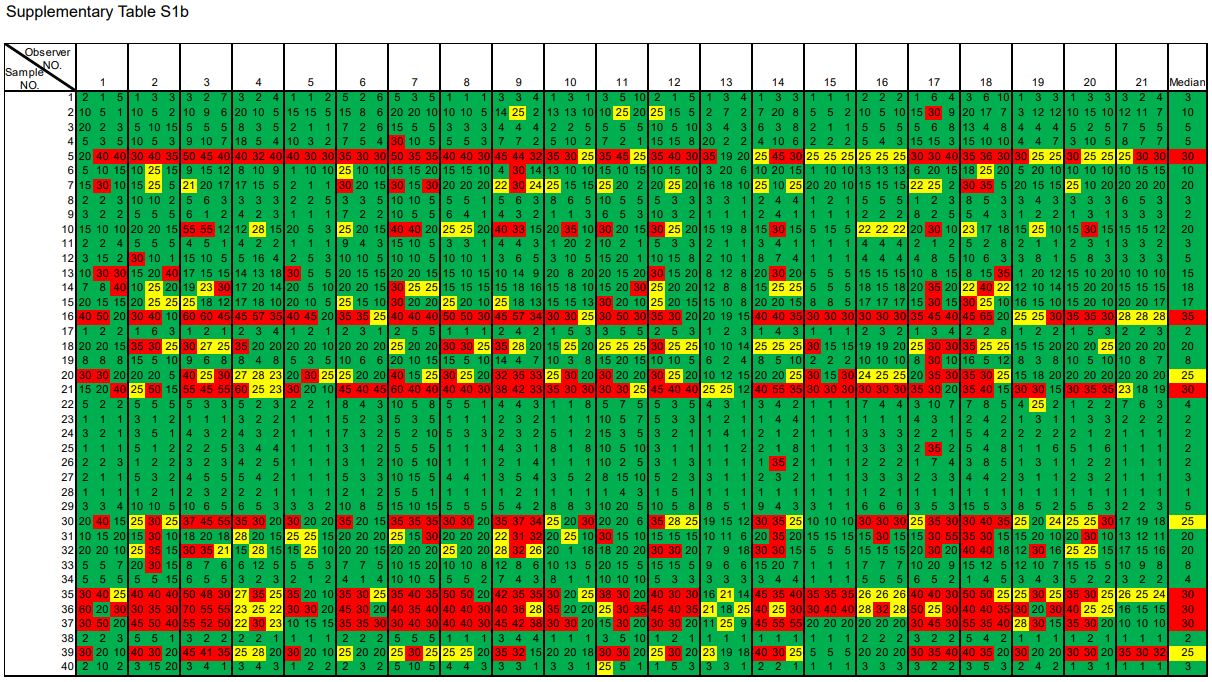

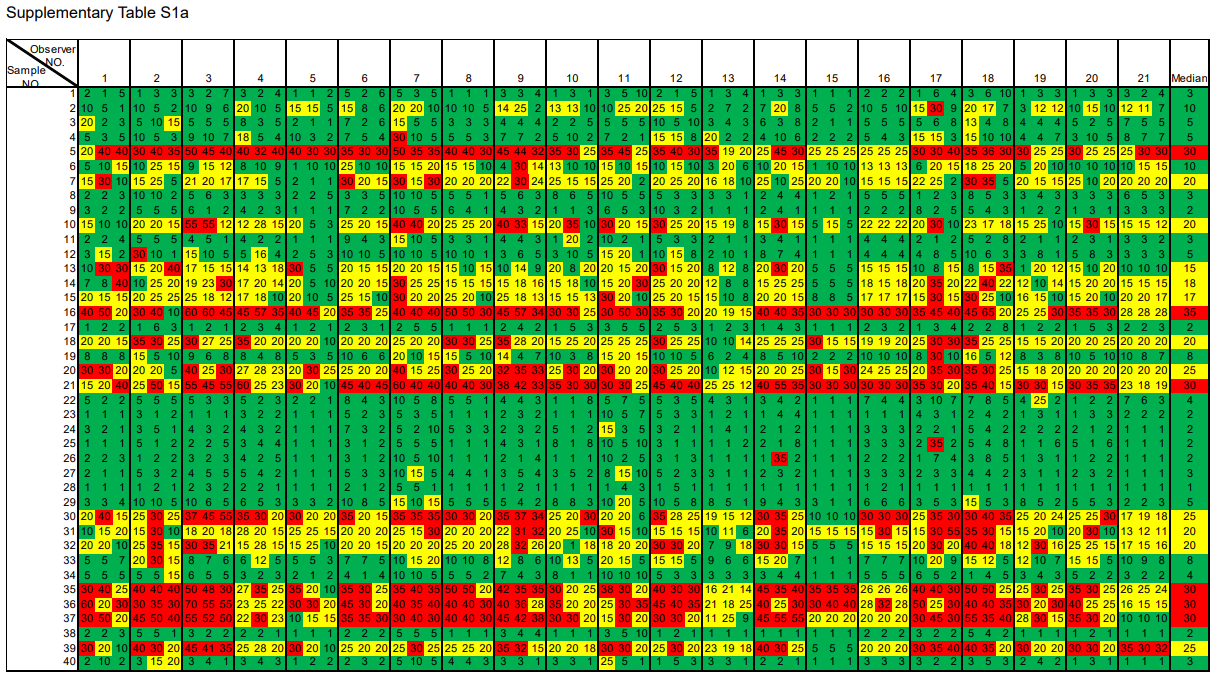


**Table S1: Heat map of KI67 scores (a: NHCC; b: NHCC9).** Rows represent sample numbers and columns represent 21 observers who evaluate IHC slices in triplicate in blind, and the median represents the median of 63 IHC scores of each sample. (a) Green color indicate that the score is equal or less than (≤)10%, yellow as between 10~30%, and red as equal or more than (≥) 30%. (b) Green color indicate that a score is equal or less (≤) 20%, yellow as 20~30%, and red as equal or more than (≥)30%.


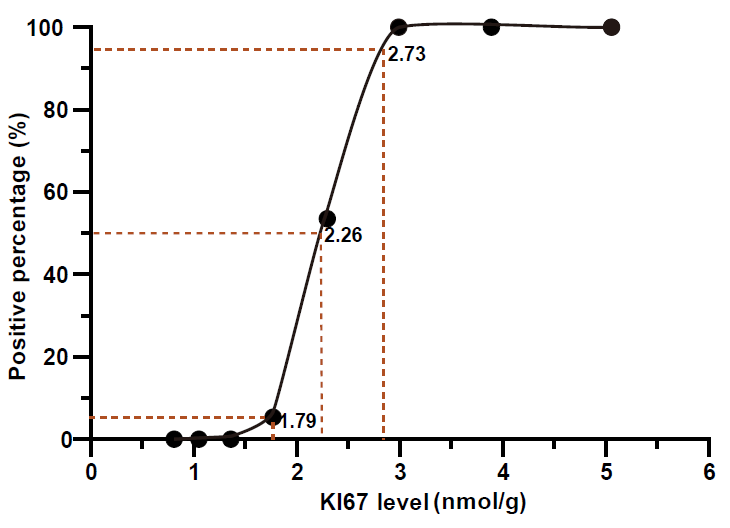


**Fig S1: Defining the C_5_, C_50_, and C_95_ of the validated 2.31 nmole/g cutoff.** The 2.31 nmol/g was developed and validated previously. Total protein lysates from several breast cancer specimens were prepared and the final concentration was adjusted around 5 nmole/g. The lysates were serially diluted and loaded in 56-plicates at indicated concentrations in the figure. C_5_, C_50_ and C_95_ of the cutoff was calculated statistically based on the percentage of specimens above 2.31 nmole/g at each concentration. The values of C_5_, C_50_ and C_95_ were marked at the figure.


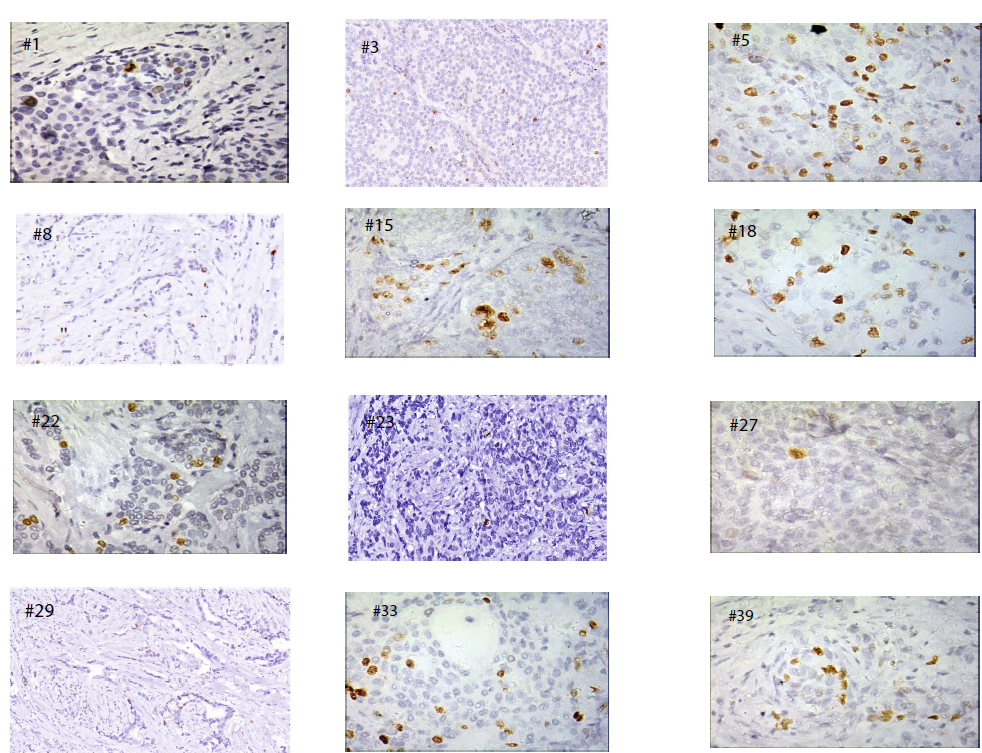


**Fig S2: Representative IHC images of 12 breast cancer specimens used in the study.** The median IHC scores and mean Ki67 levels from QDB analysis were as following (IHC vs QDB results): #1(3% vs 1.43 nmole/g); #3(5% vs 1.44 nmole/g); #5(30% vs 8.30 nmole/g); #8(3% vs 1.34 nmole/g); #15(17% vs 2.02 nmole/g); #18(20% vs 4.73 nmole/g); #22(4% vs 1.38 nmole/g); #23(2% vs 5.52 nmole/g); #27(3% vs 1.50 nmole/g); #29(5% vs 3.25 nmole/g); #33(8% vs 1.37 nmole/g); #39(25% vs 7.04 nmole/g).
